# Discovering novel reproductive genes in a non-model fly using *de novo* GridION transcriptomics

**DOI:** 10.3389/fgene.2022.1003771

**Published:** 2022-12-07

**Authors:** Mrinalini Walter, Nalini Puniamoorthy

**Affiliations:** Department of Biological Sciences, National University of Singapore, Singapore, Singapore

**Keywords:** gene expression, GridION, Illumina, novel gene, Oxford Nanopore Technologies (ONT), reproduction, *Sepsis punctum*, sexual selection

## Abstract

Gene discovery has important implications for investigating phenotypic trait evolution, adaptation, and speciation. Male reproductive tissues, such as accessory glands (AGs), are hotspots for recruitment of novel genes that diverge rapidly even among closely related species/populations. These genes synthesize seminal fluid proteins that often affect post-copulatory sexual selection—they can mediate male-male sperm competition, ejaculate-female interactions that modify female remating and even influence reproductive incompatibilities among diverging species/populations. Although *de novo* transcriptomics has facilitated gene discovery in non-model organisms, reproductive gene discovery is still challenging without a reference database as they are often novel and bear no homology to known proteins. Here, we use reference-free GridION long-read transcriptomics, from Oxford Nanopore Technologies (ONT), to discover novel AG genes and characterize their expression in the widespread dung fly, *Sepsis punctum*. Despite stark population differences in male reproductive traits (e.g.: Body size, testes size, and sperm length) as well as female re-mating, the male AG genes and their secretions of *S. punctum* are still unknown. We implement a *de novo* ONT transcriptome pipeline incorporating quality-filtering and rigorous error-correction procedures, and we evaluate gene sequence and gene expression results against high-quality Illumina short-read data. We discover highly-expressed reproductive genes in AG transcriptomes of *S. punctum* consisting of 40 high-quality and high-confidence ONT genes that cross-verify against Illumina genes, among which 26 are novel and specific to *S. punctum*. Novel genes account for an average of 81% of total gene expression and may be functionally relevant in seminal fluid protein production. For instance, 80% of genes encoding secretory proteins account for 74% total gene expression. In addition, median sequence similarities of ONT nucleotide and protein sequences match within-Illumina sequence similarities. Read-count based expression quantification in ONT is congruent with Illumina’s Transcript per Million (TPM), both in overall pattern and within functional categories. Rapid genomic innovation followed by recruitment of *de novo* genes for high expression in *S. punctum* AG tissue, a pattern observed in other insects, could be a likely mechanism of evolution of these genes. The study also demonstrates the feasibility of adapting ONT transcriptomics for gene discovery in non-model systems.

## 1 Introduction

Insects are hyper-diverse not only in terms of species richness, but also in many aspects of their reproductive biology. Most female insects mate multiple times and possess specialized organs for long-term sperm storage. This sets the stage for post-copulatory sexual selection that involves complex morphological and biochemical interactions between sperm, seminal plasma, and the female. There can also be interactions between ejaculates from competing males as they vie for limited storage space and for fertilization. Such sperm competition often leads to the evolution of extraordinarily diverse reproductive traits, including exaggerated sperm morphologies as well as rapidly diversifying seminal fluid compositions (Lupold et al., 2016; Gasparini et al., 2020; Simmons and Wedell, 2020). Some seminal fluid proteins and genes are known to evolve rapidly even among closely related species of insects (Swanson et al., 2001; Mueller et al., 2005; Findlay et al., 2008; Goenaga et al., 2015; Abry et al., 2017; Bayram et al., 2017; Bayram et al., 2019; Mrinalini et al., 2021), and they can play a crucial role in post-copulatory sexual selection in the female by forming mate plugs, affecting differential sperm storage, and even influencing female receptivity to remating (Avila et al., 2011; Wigby et al., 2020). Rapid divergence in genes underlying ejaculate-female interactions among populations can result in barriers to gene flow that generate reproductive incompatibilities and mediate incipient speciation within a species (Goenaga et al., 2015; Yamane et al., 2015).

**FIGURE 1:**
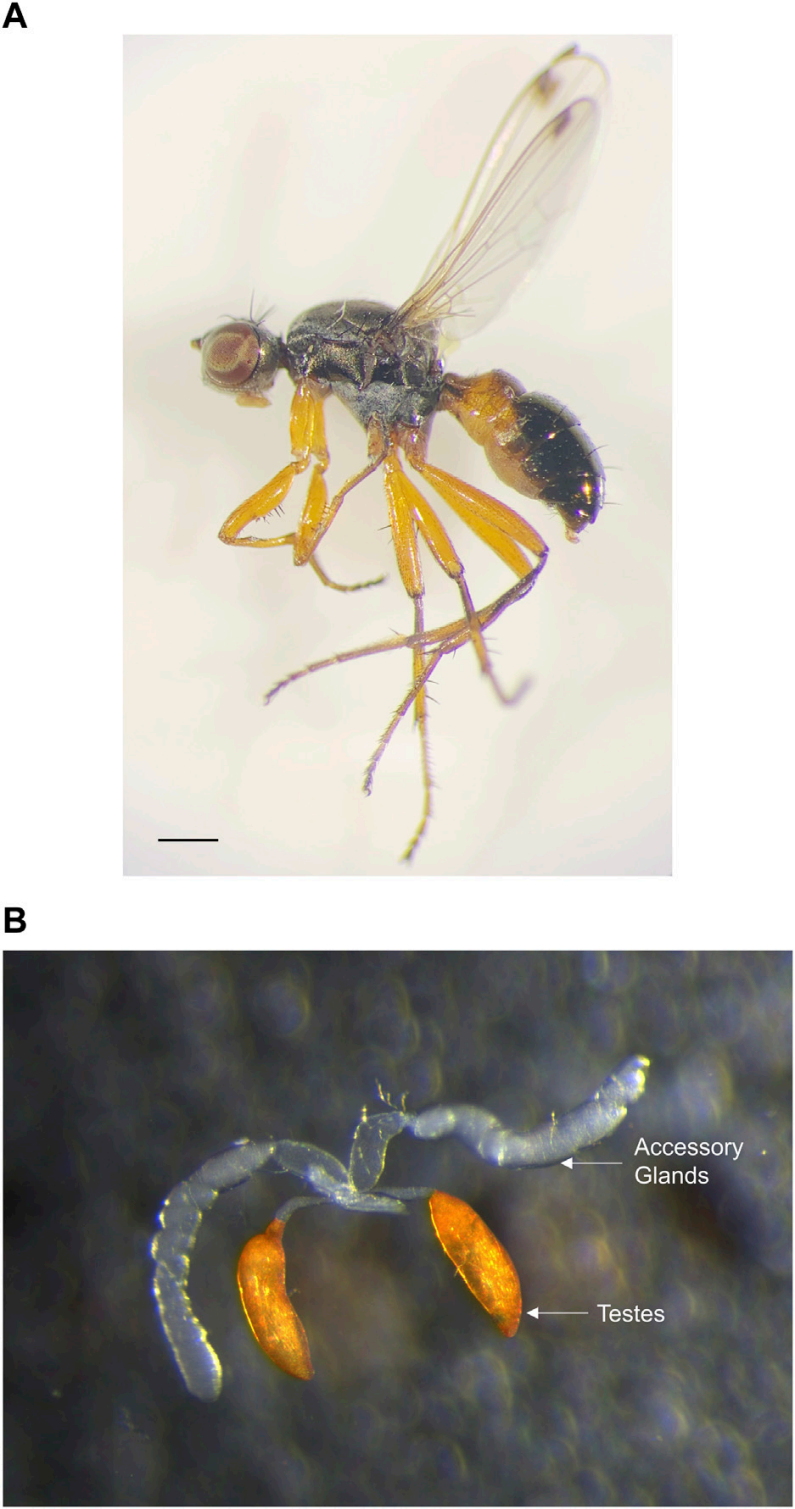
**(A)** Habitus of a male *Sepsis punctum* (1 mm scale bar) **(B)** Testes and accessory glands. Sepsid males can be easily distinguished from females based on foreleg modifications, which are absent in the latter. Males possess paired accessory glands that are translucent and are closely associated with the testes that are pigmented. Accessory glands of insects are hotspots for the recruitment of novel, species-specific reproductive genes, and this underpins the divergence of male ejaculatory proteins that play a crucial role in post-copulatory sexual selection.

The male reproductive tissues of insects typically consist of testes that are responsible for sperm production and closely associated accessory glands (AGs) that synthesize seminal fluid proteins (Figure 1). Analyses of several insect AG secretions have revealed the presence of protease inhibitors, c-type lectins, cysteine-rich secretory proteins, prohormones, antimicrobial proteins, as well as small peptides (Avila et al., 2011; Bayram et al., 2017; Mrinalini et al., 2021). However, the most striking aspect of insect AG secretions is the ubiquitous presence of dozens of newly-evolved proteins that show no similarity to proteins from other organisms and whose functions are largely unknown (Parthasarathy et al., 2009; Bayram et al., 2017; Bayram et al., 2019; Mrinalini et al., 2021). Furthermore, AG proteins are known to evolve rapidly even at the primary sequence level (Findlay and Swanson, 2010; Avila et al., 2011). Many studies on AG secretions have focused on model systems with well-characterized reference genomes like *Drosophila melanogaster,* disease vectors like *Aedes* mosquitoes, as well as agricultural pests like *Tribolium castaneum* and *Callosobruchus maculatus* beetles (Parthasarathy et al., 2009; Avila et al., 2011; Goenaga et al., 2015; Yamane et al., 2015; Ahmed-Braimah, 2016; Sayadi et al., 2016; Bayram et al., 2017; Bayram et al., 2019; Degner et al., 2019; Wigby et al., 2020). A handful of other insect groups that include crickets, moths, bees, and ants have also been explored (Braswell et al., 2006; Gorshkov et al., 2015; Fuessl et al., 2018; Gotoh et al., 2018; Saraswathi et al., 2020), but overall, studies on non-model insect reproductive gene discovery are still sparse. In a recent study on dung beetle, we discovered that AGs are hotspots for recruitment of completely novel genes for reproductive function whilst the testes generally express more conserved genes involved in sperm production (Mrinalini et al., 2021). We found that rapid evolution at the genomic level, driven by the birth of novel genes and their subsequent recruitment for high-expression in the AGs, underpins the starkly divergent reproductive AG gene repertoires even in closely-related dung beetle species (Mrinalini et al., 2021).

While early studies on insect AGs were limited to investigations of single AG genes and their gene products, recent advances in high-throughput transcriptomics and proteomics have facilitated the discovery of AG transcripts and proteins on a much larger scale (Wei et al., 2015; Sayadi et al., 2016; Bayram et al., 2017; Gotoh et al., 2018; Bayram et al., 2019; Saraswathi et al., 2020; Wigby et al., 2020; Mrinalini et al., 2021). *De novo* RNA sequencing (RNAseq) transcriptomics has allowed us to explore transcript libraries from any non-model species, even in the absence of a reference genome, because these technologies do not require *a priori* sequence knowledge. Among several short-read technologies developed for *de novo* RNAseq, Illumina is an established market leader due to its high base calling accuracy (>99.9%), high data yield, and the availability of well-established bioinformatics tools and best practices for data analysis (Conesa et al., 2016; Hölzer and Marz, 2019; Corchete et al., 2020). Third Generation long-read sequencing of transcriptomes, such as PacBio and Oxford Nanopore Technologies (ONT), eliminate the need for contig assembly and offer many advantages including long reads (>10 kbp), end-to-end transcripts, structural variants, isoform-level resolution of genes and expression. ONT long-read transcriptomics have proven useful for *de novo* and reference-based transcript sequencing and expression analysis in both model and non-model organisms (Hargreaves and Mulley, 2015); (Weirather et al., 2017; Bayega et al., 2018; Sessegolo et al., 2019; Soneson et al., 2019; Workman et al., 2019; Halstead et al., 2021; Lin et al., 2021). Despite these advances, discovering novel AG genes in non-model species can still be a challenge due to difficulties arising from lack of genomic/transcriptomic reference databases, the presence of novel species-specific genes, and the complete lack of protein homology to any other species.

In this study, we investigate reproductive genes and quantify gene expression in the AGs of a non-model dung fly species, *Sepsis punctum* (Diptera; Sepsidae). This is an ecologically relevant insect, often found on decaying organic material such as cattle dung and is widespread across North America and Europe. Sepsid flies are emerging study systems in a range of disciplines such as eco-toxicology (Blanckenhorn et al., 2013a; Blanckenhorn et al., 2013b), biogeography (Giesen et al., 2017; Giesen et al., 2019; Blanckenhorn et al., 2021), evo-devo (Herath et al., 2015) as well as reproductive biology (Puniamoorthy et al., 2008; Puniamoorthy et al., 2009). In particular, *S. punctum* populations in North America and Europe differ significantly with respect to mating behaviour as well as male reproductive investments and female remating frequencies, making it an interesting model for sexual selection studies (Puniamoorthy et al., 2012a; Puniamoorthy et al., 2012b; Rohner et al., 2016; Blanckenhorn et al., 2021). However, with the exception of species from the genus *Themira,* which is distantly-related to *S. punctum*, there is generally a lack of genic, genomic, or transcriptomic data for sepsid species. Here, we use reference-free GridION transcriptomics, an ONT long-read transcriptomics method, to characterize AG genes and quantify their expression in *S. punctum.* We implement a *de novo* ONT transcriptome pipeline with error correction procedures, and we evaluate gene sequence and gene expression results from this emerging technology against high-quality Illumina short-read data.

## 2 Materials and methods

### 2.1 Dissection of accessory glands from *Sepsis punctum*

The sampling and maintenance of *S. punctum* cultures followed previously published work (Puniamoorthy et al., 2012a; Rohner et al., 2016). For this study, a North American population from Ottawa (45.43 °N, −75.67 °E) was used and adult flies were housed in plastic containers measuring 11 cm by 9 cm by 9 cm and reared at a temperature of 26°C with cattle dung, sugar, and water given *ad libitum*. A mixture of mated and unmated male flies that were four to 10 day old were aspirated from culture containers into plastic vials, cooled at −20°C for 10 min, and placed on ice until dissection. Each fly was transferred to a glass slide and the abdomen was dissected into 1X PBS. Paired accessory glands were isolated and collected into 1.5 ul microcentrifuge tube snap frozen on dry ice. For the purposes of this study, we excluded the testes, ejaculatory bulb, ejaculatory duct, and aedeagus. For ONT GridION, tissues from 80 flies were pooled to allow for protocol optimisation, and for Illumina HiSeq, tissues from 63 flies were pooled. For each sequencing technology two biological replicates of pooled tissues were collected and samples were stored at−80°C until RNA extraction.

### 2.2 RNA extraction

Total RNA was extracted using Aurum Total RNA Mini Kit (BIO-RAD Cat # 732–6820)*.* Samples stored at −80**°**C were centrifuged at 13,000 rpm for 20 min at 4**°**C and placed on ice. 700 ml lysis solution was added to each sample and homogenized using PTFE pestles. The lysate was centrifuged for 3 min at 4**°**C and the supernatant was transferred to new tube. 700 ul of 60% ethanol was added and thoroughly mixed by vortexing for 2–3 min to make sure there was no visible bilayer. 700 ul of homogenized lysate was transferred into an RNA binding column inserted into a wash tube and the set up was centrifuged for 1 min. The filtrate was discarded, and the same wash was repeated a second time. 700 ul of low stringency wash was added to column and centrifuged for 1 min and filtrate discarded. 80 ul of DNase (5ul of DNase I solution +75 ul of DNase solution) was added to each column and incubated at room temperature for 25 min. The samples were washed two more times, first with 700 ul of high stringency wash solution and second with 700 ul of low stringency wash with centrifuging for 1 min and discarding of filtrate after each wash. The samples were spun for 3 min to remove residual wash solution and the RNA binding column was transferred to 1.5 ul microcentrifuge tubes. 40 ul of elution solution was added to the membrane of the binding column and after 1 minute of membrane saturation, the sample was centrifuged for 2 min to elute total RNA.

### 2.3 cDNA library preparation and RNAseq

#### 2.3.1 ONT GridION

ONT GridION offers Direct RNA or Direct cDNA library preparation and sequencing options, however these technologies require high amounts of starting RNA input. The RNA quantities of our samples were inherently low given the small size of our study species *S. punctum* (2–7 mm in length) and even smaller size of reproductive tissues (Figure 1). Therefore, the PCR-cDNA (PCB109) protocol was used, which allows for lower RNA input. Total RNA samples were submitted to Genome Institute of Singapore, Singapore, for ONT GridION long-read RNAseq. Nanodrop 2000 spectrophotometer (NanoDrop, Wilmington, DE) was used to determine RNA concentration, and quality check was performed using Agilent RNA Screentape kit with Agilent Tapestation 4200 (Agilent, Santa Clara, CA). 100 ng total RNA was used for cDNA synthesis and strand switching of full-length poly AAA tail. cDNA was amplified with 5’ barcoded primers and sequencing adapter annealing. Thirteen cycles of amplification were performed allowing an extension time of 10 min to amplify transcripts up to 12 kbp. Barcoded libraries were multiplexed by pooling at 100 fmol based on average Agilent DNA 12000 size, and sequencing was performed on one FLO-MIN106D (R9.4.1) GridION flowcell. Guppy v4.0.11 was used in high accuracy (hac) mode to perform live basecalling on GridION.

#### 2.3.2 Illumina HiSeq

For Illumina short-read sequencing, total RNA was shipped to Genomics Research Center at University of Rochester, New York, for cDNA library preparation and sequencing. Total RNA concentration was determined with NanoDrop 1000 spectrophotometer (NanoDrop, Wilmington, DE) and RNA quality assessed with the Agilent Bioanalyzer (Agilent, Santa Clara, CA). TruSeq RNA Sample Preparation Kit V2 was used for library construction as per manufacturer’s protocols. Briefly, mRNA was purified from 100 ng total RNA with oligo-dT magnetic beads and then fragmented. First-strand cDNA was synthesized with random hexamer priming followed by second-strand cDNA synthesis. End repair and 3′ adenylation was performed on the double stranded cDNA. Illumina adaptors were ligated to both ends of the cDNA, purified by gel electrophoresis and amplified with Polymerase Chain Reaction (PCR) primers specific to the adaptor sequences to generate amplicons of approximately 200–500 bp in size. Libraries were loaded at a concentration of 8 p.m. per lane and Paired End reads of length 125 bp were sequenced on HiSeq 2500 v4 platform.

### 2.4 *De novo* transcriptome pipelines and gene expression analysis

**FIGURE 2:**
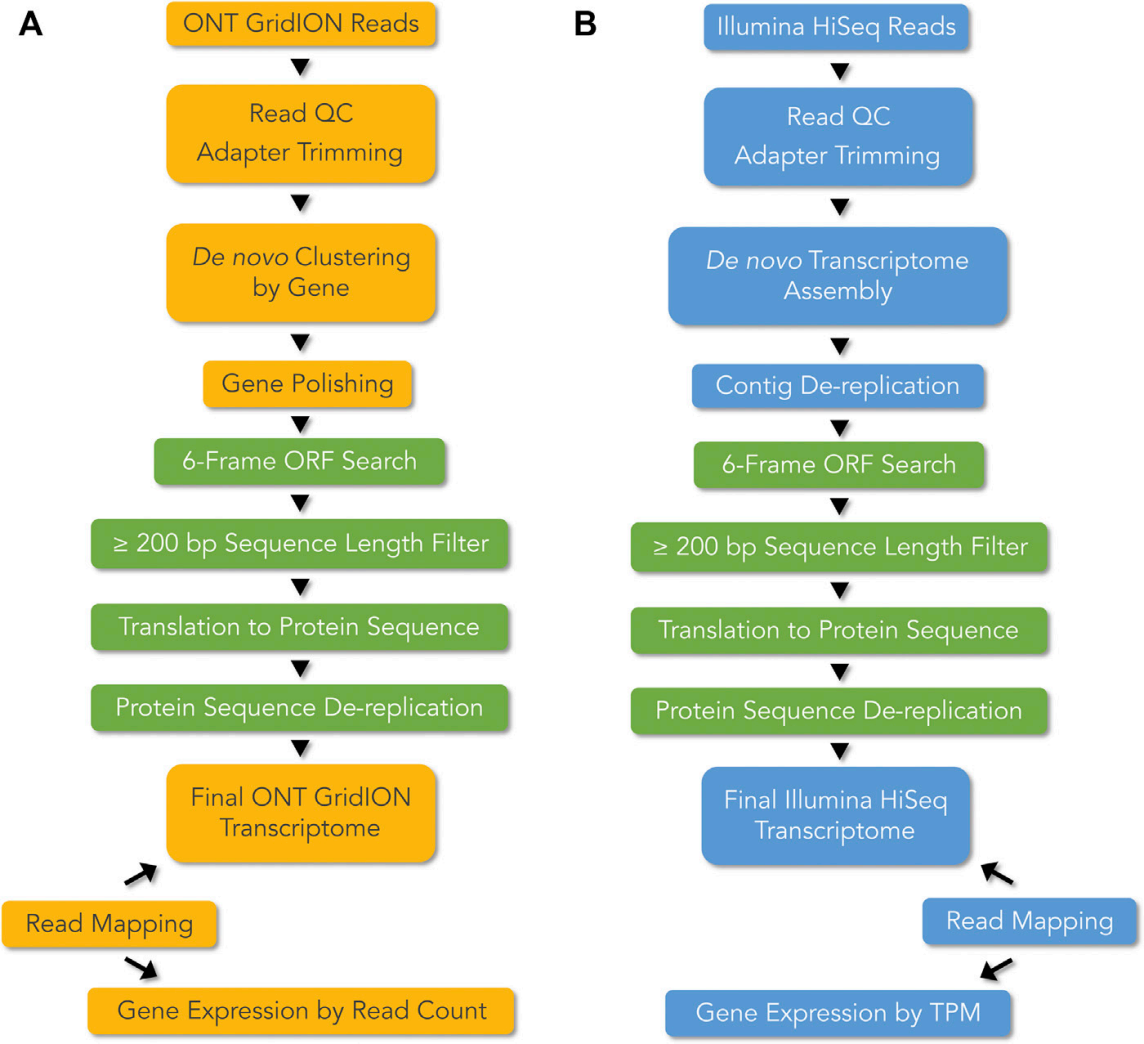
*De novo* transcriptome pipelines for **(A)** ONT long-read technology, and **(B)** Illumina short-read technology. mRNA from the accessory glands of *Sepsis punctum*, was used for cDNA library preparation and RNAseq using ONT long-read and Illumina short-read technologies. ONT transcripts were generated by *de novo* gene clustering, consensus generation, and gene polishing, whereas for Illumina short-reads were *de novo* assembled to reconstruct contigs. Preliminary transcriptomes were filtered using common steps (shown in green), using six-frame Open Reading Frame (ORF) searches, ≥ 200 bp length, and dereplication at 100% protein identity, to derive final transcriptomes. Gene expression was quantified using read count as a proxy for ONT and Transcript per Million (TPM) for Illumina.

Two different bioinformatics pipelines were employed, with some steps commonly implemented in both pipelines, for *de novo* transcriptome construction and analysis of ONT long-reads and Illumina short-reads (Figures 2A,B). For ONT, *de novo* gene clustering, consensus sequence calling, and gene polishing were used to derive error corrected gene sequences (Sahlin and Medvedev, 2019; Sahlin et al., 2021). For Illumina, a *de novo* transcriptome assembly approach was used to reconstruct contigs from which full length coding DNA sequences (CDS) could be derived. The two methods differed in gene expression quantification in that, read count was used as a proxy for gene expression in the case of ONT, whereas Transcript per Million (TPM) was used for Illumina based gene expression calculation (Figure 2).

### 2.5 *De novo* ONT pipeline

A suite of bioinformatics tools, including standalone tools and those developed by ONT, were used for *de novo* transcriptome analysis of ONT long-reads. Read quality filtering, orientation, and trimming was performed using *Pychopper v2* (https://github.com/nanoporetech/pychopper) with default parameters, and read statistics were analysed using *NanoPlot 1.32.1* (De Coster et al., 2018). A non-hybrid approach of using ONT long-reads for error correction was employed, as our ONT and Illumina read data were derived from separate biological samples. Error correction of each ONT transcriptome was performed using ONT long-reads within the same biological sample. *IsONclust2,* implemented in *pipeline-nanopore-denovo-isoforms* (https://github.com/nanoporetech/isONclust2) was used for *de novo* clustering of ONT long-reads and one sequence cluster was generated for each gene (Sahlin and Medvedev, 2019; Sahlin et al., 2021). Consensus sequences were called for each sequence cluster to generate one consensus sequence per gene. The consensus gene sequences were further polished using raw reads in *medaka 1.2.5* (https://github.com/nanoporetech/medaka). Open Reading Frames (ORFs) were derived by translating polished sequences in all six frames using *getorf* provided with *EMBOSS:6.6.0* (https://www.bioinformatics.nl/cgi-bin/emboss/getorf). ORF sequences ≥200 bp were translated into protein sequences using *transeq* provided with *EMBOSS:6.6.0* (https://www.bioinformatics.nl/cgi-bin/emboss/transeq), and a final dereplication was performed at 100% protein sequence identity using *CD-HIT v4.7* (Li and Godzik, 2006; Fu et al., 2012). For gene expression quantification, long-reads were mapped back to filtered *de novo* ONT transcriptomes using *minimap2,* excluding any secondary alignments (Li, 2018). S*amtools 1.7* (Li et al., 2009) was used to further filter aligned reads, with any supplementary and secondary alignments discarded. Only reads aligning on ≥ 80% of their length were counted towards gene expression quantification.

### 2.6 *De novo* Illumina pipeline

Raw reads were processed in *Trimmomatic-0.36* (Bolger et al., 2014) for adapter removal and quality trimming. A sliding window quality score cut-off of Q30 was applied and reads of minimum 100 bp in length were retained. For each sample, cleaned reads were *de novo* assembled into contigs using *Trinity v2.8.6* (Grabherr et al., 2011), and the resulting contigs were de-replicated at 100% identity at nucleotide level using *CDHIT 4.7* (Li and Godzik, 2006; Fu et al., 2012). The remaining contigs were translated in all six frames to search for ORF prediction using *getorf* (*EMBOSS:6.6.0*) (https://www.bioinformatics.nl/cgi-bin/emboss/getorf), and all sequences containing ORFs of ≥200 bp were retained. The contigs with ORFs were translated into protein sequences using *transeq* (*EMBOSS:6.6.0*) (https://www.bioinformatics.nl/cgi-bin/emboss/transeq), and a final dereplication was performed at 100% protein sequence identity. Reads were mapped back to the filtered transcriptome assembly using an alignment-free method in *salmon v1.0.0* (Patro et al., 2017) to generate TPM values that represent gene expression.

### 2.7 Sequence curation and gene orthology

*Sepsis punctum* lacks species-specific reference databases to compare our *de novo* transcriptome constructions. While genomic/transcriptomic data are available for *Themira sp.,* it is not the ideal species for verifying the accuracy of *S. punctum* AG transcripts because it is from a basal, distantly related genus and insect AG genes and protein compositions vary at species and even population levels (Swanson et al., 2001; Goenaga et al., 2015; Abry et al., 2017; Bayram et al., 2019; Mrinalini et al., 2021). Therefore, building on our *de novo* approach transcriptomics, a reference-free approach was taken for transcript curation and an extensive manual curation of *S. punctum* AG genes was performed.

A gene expression cut-off was applied, and the top 100 highest expressed transcripts were selected from each of the four *de novo* transcriptomes (ONT-1, ONT-2, ILL-1, ILL-2) since transcriptome-based quantification of gene expression generally shows a steep drop after the first few transcripts. The subset of 400 sequences was further examined to filter out chimeric and contaminant bacterial and nematode sequences with BLASTX in *nr* using *DIAMOND v 0.8* (Buchfink et al., 2014; Buchfink et al., 2021). Using the cleaned sequence set from each sample, putatively orthologous genes were established in the *de novo* transcriptomes of the remaining three samples by a reciprocal BLASTP with an *e-value* cut-off of 1e-5. These putative orthologs were further curated by manually examining end-to-end alignments. Finally, a set of 40 high-confidence and high-quality accessory gland genes were derived with orthologs established in all four samples and used for the downstream analyses.

### 2.8 Evolutionary novelty and functional classification of curated genes

To examine whether these 40 genes included novel AG genes that have evolved in *S. punctum*, we used a two-step approach: First, we conducted BLASTP and BLASTX searches against *nr* that contains protein sequences from broad taxonomic categories; and second, using BLASTN and TBLASTX against the genome of the closest sepsid relative, *Themira minor* (GenBank Accession No. GCA_001014575.1), to potentially identify unannotated genic regions in genomic scaffolds*.* A cut-off *e-value* of 1e-5 and at least 50% identity over 70% of the sequence was applied to call BLAST hits. Genes with no hits to both *nr* and *Themira minor* genome were defined as novel genes that have *de novo* evolved in *S. punctum*. Functional annotation was also performed for the 40 genes and broad functional categories were assigned based on BLASTP results against *nr*. Secretory proteins were identified by translating coding DNA sequences in MEGAX and analysing the resulting proteins in SignalP 5.0 webserver (https://services.healthtech.dtu.dk/service.php?SignalP-5.0) to check for the presence of signal peptides.

### 2.9 Evaluation of ONT transcriptome

ONT long-reads are prone to high error rates, therefore the usefulness of our *de novo* ONT transcriptomics pipeline and error correction procedures in mitigating the effects of sequencing errors was evaluated (Figure 2A). Given that our study is reference-free, and Illumina short-reads are of high quality, a sequence similarity analysis was performed by comparing ONT gene sequences to Illumina gene sequences. The Illumina sample, ILL-1, was designated as the control sample and the remaining samples ONT-1, ONT-2, as well as ILL-2 were the test samples. Comparing ILL-2 to ILL-1 control sample allowed for within-Illumina assessment that can uncover effects of tissue pooling and natural genetic variation in the population. Sequence similarity values were generated by performing BLASTN of nucleotide sequences and BLASTP of translated protein sequences from 40 genes of three test samples against 40 genes from ILL-1 control. Percent sequence similarities were summarized, including median sequence similarity and percentage of sequences with 100% match to ILL-1 control. Similarities of all 40 genes were plotted as a heatmap for visualization at individual gene level.

## 3 Results

### 3.1 *De novo* transcriptome statistics

After implementing two separate *de novo* transcriptome pipelines for ONT and Illumina read data (Figure 2), we generated summary statistics for the transcriptomes. Table 1 provides the statistics for experimental details, cDNA library preparation, RNAseq reads, and filtered *de novo* transcriptomes.

**TABLE 1:** Statistics from ONT long-read and Illumina short-read cDNA library preparation, RNAseq reads filtering, and final *de novo* transcriptomes.

	ONT		Illumina	
	ONT-1	ONT-2	ILL-1	ILL-2
**Experimental Details**				
No. of pooled individuals	80	80	63	63
Total RNA used for library prep (ng/ul)	100	100	100	100
RNAseq Read Statistics				
No. of Reads (Million)	4.48	5.28	29.64	32.96
Total No. of Bases (Gbp)	2.05	2.14	7.41	8.24
Max. Read Length (bp)	11,837	9,370	125	125
Mean Read Length (bp)	456	405	125	125
Min. Read Length (bp)	58	50	125	125
Mean Read Quality	11.7	11.6	35.49	35.52
GC Content (%)	39.5	39.2	46	46
**Filtered *De Novo* Transcriptome Assembly Statistics**				
No. of Transcripts	44,958	33,564	44,523	45,681
Largest Transcript (bp)	4,422	2,964	13,932	12,372
Mean Transcript Length (bp)	358	342	513	492
GC Content (%)	54.3	53.9	54.4	53.7

### 3.2 RNAseq read statistics

ONT long-read sequencing generated 4.48 M and 5.28 M reads for samples ONT-1 and ONT-2 respectively, whereas Illumina sequencing generated 29.64 M and 32.96 M reads for ILL-1 and ILL-2 respectively. Long-read lengths range from 50 bp to a maximum of 11,837 bp for ONT-1 and 50 bp to 9,370 bp for ONT-2. Average read quality, represented by Phred scores, are 11.7 and 11.6 for ONT-1 and ONT-2 respectively. For Illumina, read qualities were much higher at 35.5 on average from the two sample. Filtered RNAseq read GC content was 39.5% and 39.2% for ONT-1 and ONT-2, whereas GC content for both Illumina samples was 46%.

**FIGURE 3:**
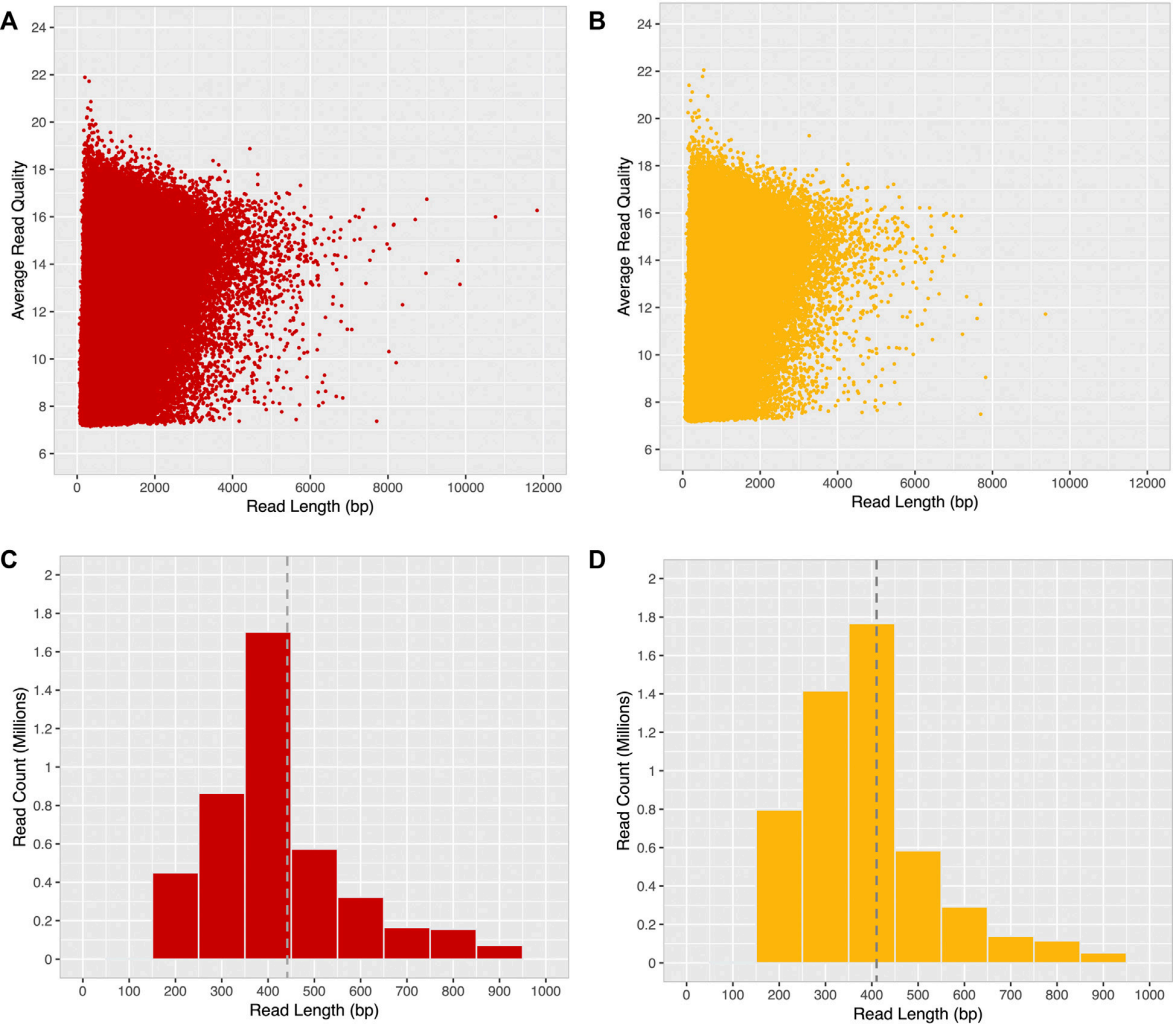
Read quality and read length distribution of filtered ONT long-reads. After a minimum base quality cut-off of 7, a majority of long-reads for both samples **(A)** ONT-1, and **(B)** ONT-2 were within the 4 kbp range, with an average base quality ≤19. Most long-reads were 400 bp in length for both **(C)** ONT-1, and **(D)** ONT-2, with N50 values of 448 bp and 407 bp represented as grey dotted lines.

After quality filtering, most long-reads were found to be distributed within the 4000 bp range, with an average base quality ≤19 (minimum base quality 7) for both ONT-1 and ONT-2 (Figures 3A,B). A histogram of read length distribution shows that the maximum number of long-reads occur in the range of 250–450 bp for both samples and read N50 were 448 bp and 407 bp for ONT-1 and ONT-2 respectively (Figures 3C,D; Table 1).

### 3.3 Filtered *de novo* transcriptome statistics

After *de novo* gene clustering, gene consensus calling, gene polishing and subsequent filtering (Figure 2A), *de novo* transcriptomes contained 44,958 and 33,564 transcripts in ONT-1 and ONT-2 respectively (Table 1). For Illumina, *de novo* transcriptome assembly followed by filtering steps showed 44,523 and 45,681 assembled contigs for ILL-1 and ILL-2 respectively (Table 1). ONT long-read transcript N50 were 363 and 345 bp, with the largest transcripts at 4,422 and 2,964 bp, for ONT-1 and ONT-2 respectively (Table 1). For Illumina short-read transcripts, both values were much higher, with N50 values at 702 and 645 bp and longest transcript lengths at 13,932 and 12,372 bp, for ILL-1 and ILL-2 respectively. Although GC content of filtered RNAseq reads varied between ONT and Illumina, the GC content of the final *de novo* transcriptomes from both technologies were comparable at c. 54% (Table 1).

### 3.4 Evolutionary and functional characterisation

**FIGURE 4:**
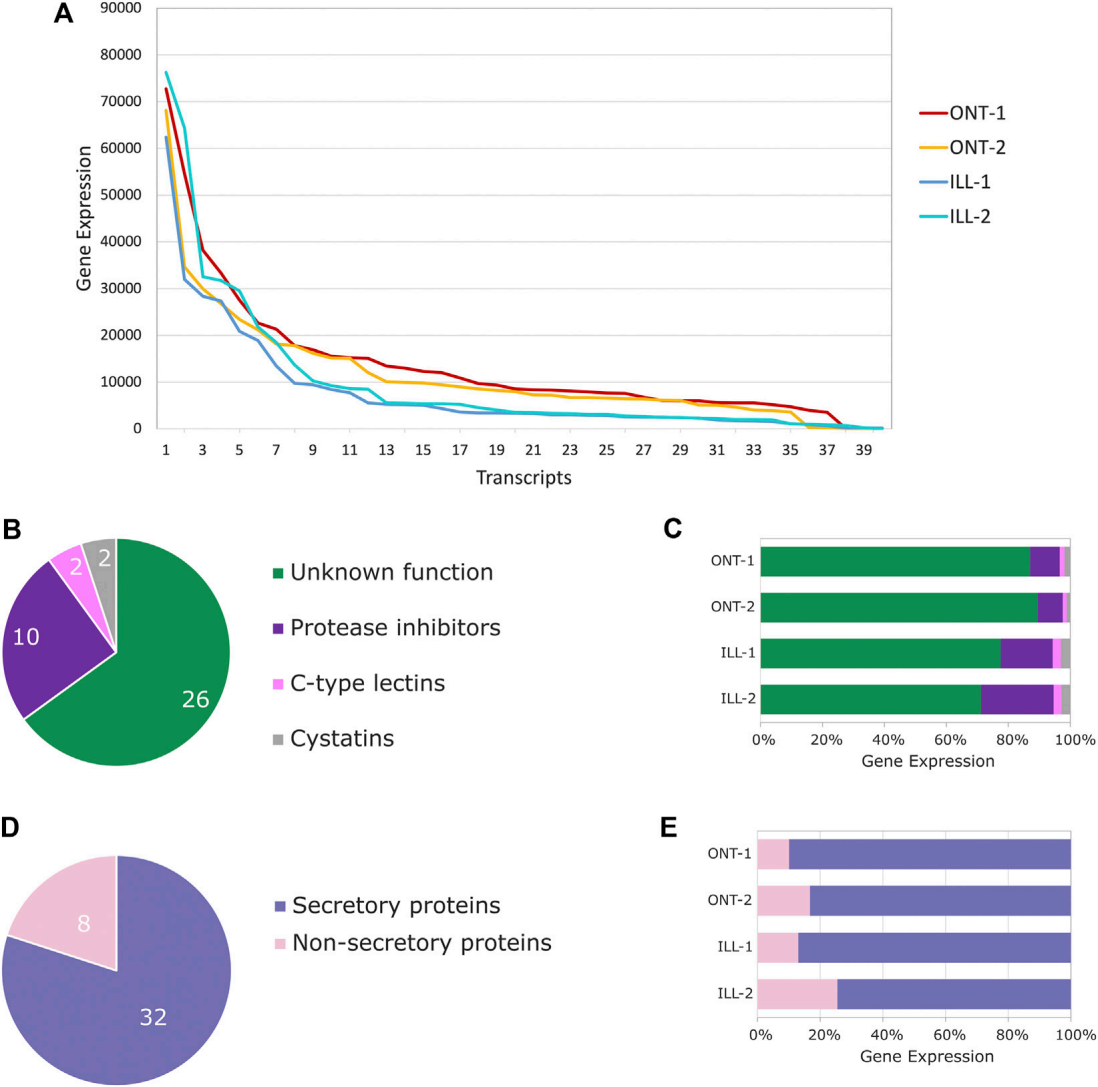
Gene expression and gene function in *S. punctum* accessory gland genes. Analysis of 40 highest expressed genes in *S. punctum* AGs showed **(A)** a high level of congruence in overall gene expression pattern between ONT read counts and Illumina Transcripts per Million (TPM) **(B)** 26 genes of *unknown function*, 10 *protease inhibitors,* and 2 each of *C-type lectins* and *cystatins,* **(C)** genes of *unknown function* were the highest expressed gene category, followed by *protease inhibitors,* in both ONT and Illumina samples **(D)** 32 genes encoding secretory proteins and eight genes encoding non-secretory proteins, and **(E)** genes encoding secretory proteins accounted for the majority of expression in both ONT and Illumina samples.

The functional aspects of *S. punctum* AG genes were characterized using gene expression analysis, BLASTP against NCBI non-redundant database (*nr*) Version 2.2.26 for functional annotation, and signal peptide analysis of the translated protein sequences (Figure 4). Ranking of accessory gland genes from high expression (1) to low expression (40) showed high level of congruence in overall gene expression pattern across all four samples despite using two different methods of quantification for the two technologies, i.e., read count for ONT samples and TPM for Illumina samples (Figure 4A). The highest expressed transcript showed read count of 72,731 and 68,120 for ONT-1 and ONT-2 respectively and TPM of 62,424 and 76,279 for ILL-1 and ILL-2 samples respectively. In both ONT and Illumina, a steep drop in gene expression occurred and expression levels tapered off from transcripts ranked at 12 and 13 and reached negligible levels by transcript 40.

Of the 40 genes analysed, 65% (26) were novel genes that evolved specifically in the genome of *S. punctum* as they did not return any hits against *nr* or against the genome of the closest sepsid relative, *Themira minor*. The remaining 14 genes were found in other insect species, some of which were interestingly identified from the male AGs of fly species as well (e.g., *Drosophila sp*.). Functional annotation showed that *S. punctum* AG genes fall into four main categories: *unknown function*; *protease inhibitors*; *C-type lectins*; and *cystatins*. Incidentally, all novel genes that *de novo* evolved in *S. punctum* also fell into the category of *unknown function* due to lack of even partial BLASTP homology in their protein sequences. However, these genes accounted for most of the gene expression in the accessory glands, i.e., 87% and 90% of total expression in ONT-1 and ONT-2 respectively, and 78% and 71% of total expression in ILL-1 and ILL2 respectively (Figure 4C). Of the remaining 14 genes, 10 genes were *protease inhibitors,* and they were the second highest expressed gene category, with 10% and 8% in ONT-1 and ONT-2, and 17% and 23% in ILL-1 and ILL-2 respectively (Figures 4B,C). Two genes each for *cystatins* and *C-type lectin* were found, and both gene families were the least expressed, with 1%–3% of total expression in both ONT and Illumina (Figures 4B,C). Sequence analysis for the presence of secretory signals showed that 80% (32) of 40 AG genes synthesize proteins that were secretory in nature (Figure 4B). Moreover, genes encoding secretory proteins also accounted for the majority AG gene expression, with 90% and 83% of total expression in samples ONT-1 and ONT-2 and 87% and 75% in samples ILL-1 and ILL-2 respectively.

**FIGURE 5:**
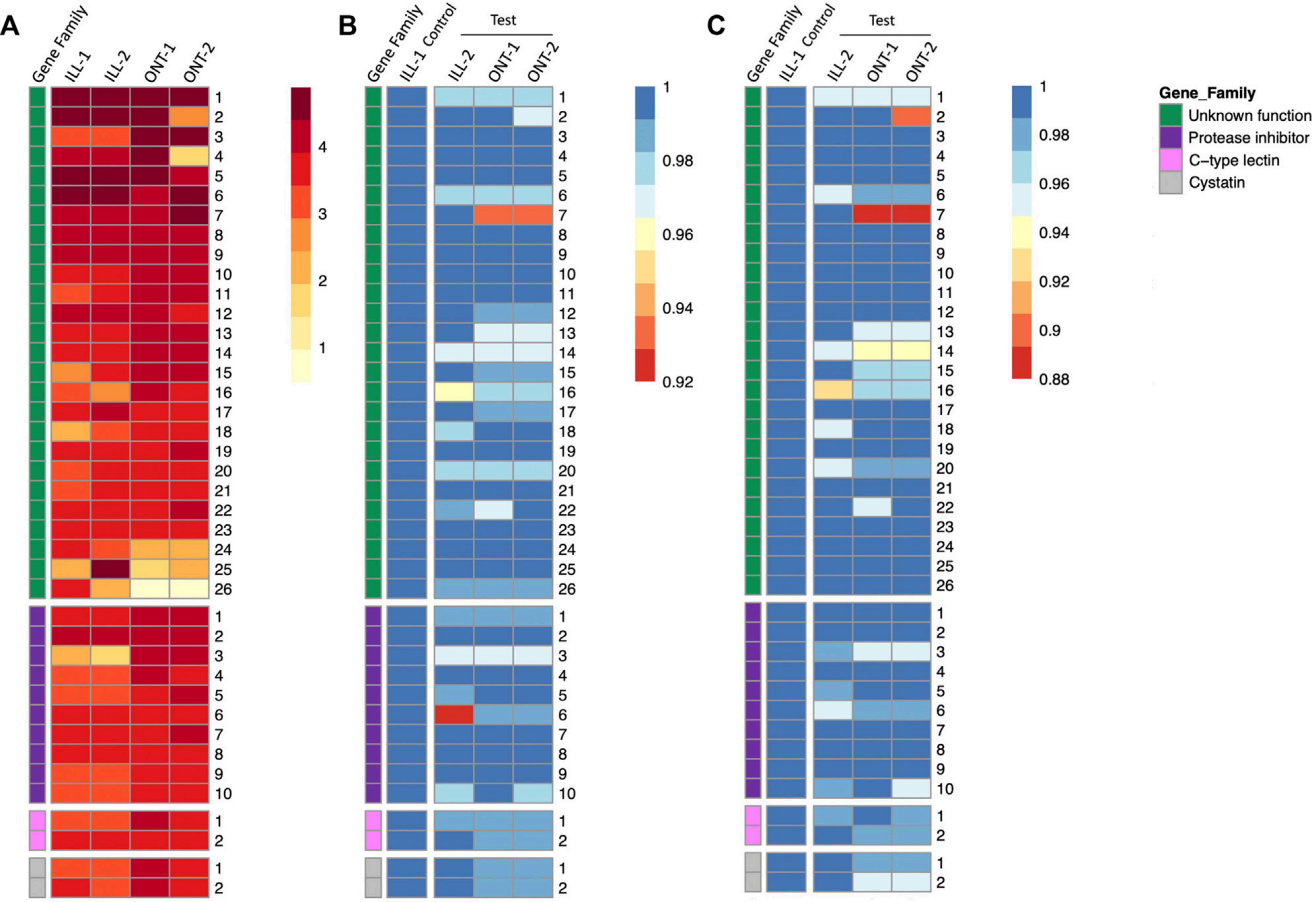
Heatmaps of gene expression and sequence similarity from 40 highest expressed accessory gland genes in *Sepsis punctum*. **(A)** Gene expression quantified by read count for ONT samples and TPM for Illumina samples and genes are grouped by functional categories. Gene of *unknown function* were among the highest expressed in both ONT and Illumina samples. Sequence similarities at **(B)** nucleotide level, and **(C)** protein level are plotted by comparing test samples ONT-1, ONT-2, and ILL-2 against a designated control sample ILL-1.

Gene expression quantification of individual genes in all the four samples are represented as a heatmap, with genes grouped by functional categories (Figure 5A). Novel genes of unknown function were among the highest expressed genes in *S. punctum* accessory glands in both ONT and Illumina samples (Figure 5A). Expression levels of several individual genes were largely comparable across samples and across sequencing technologies, with some variation between and even within technologies (Figure 5A).

### 3.5 Evaluation of ONT transcriptomes

Summary statistics of nucleotide and protein sequence similarities within each technology and comparing GridION samples (ONT-1 and ONT-2) against a representative Illumina control sample (ILL-1) are shown in Table 2. Median sequence similarities within each technology were congruent with each other, with Illumina showing 99.65% and 99.31% at nucleotide and protein levels respectively and GridION showing 100% for both. Median sequence similarities across technologies were comparable to within technology values as well. The two GridION test samples ONT-1 and ONT-2, when compared against Illumina control, showed median sequence similarities of 99.60% and 99.53% at nucleotide level and 98.99% and 98.93% at protein level respectively.

**TABLE 2:** Summary statistics of sequence similarity at nucleotide and protein level. Sequence similarities were generated within each technology and across technologies by comparing GridION test samples (ONT-1 and ONT-2) against a representative Illumina control sample (ILL-1).

	Within technology		Illumina control v/s GridION	
	ONT	Illumina	ONT-1	ONT-2
**Nucleotide Level**				
Median sequence similarity of 40 genes	100.00%	99.65%	99.60%	99.53%
Sequences with 100% similarity	93%	65%	53%	50%
**Protein Level**				
Median sequence similarity of 40 genes	100.00%	99.31%	98.99%	98.93%
Sequences with 100% similarity	90%	43%	43%	45%

In GridION, over 90% of both nucleotide and protein sequences showed 100% similarity across the two samples. In contrast, a much lower proportion of sequences with 100% similarity were seen among the two Illumina samples, with 65% among nucleotide sequences and 43% among protein sequences. Further, 53% and 50% of GridION sequences were 100% similar to Illumina control at nucleotide level, whereas 43% and 45% of GridION sequences were 100% similar to Illumina control proteins. Sequences similarities of individual genes are represented as heatmaps in Figures 5B,C. No trend is observed within or among the four functional categories, i.e., novel genes, *protease inhibitors, C-type lectins,* and *cystatins.*

## 4 DISCUSSION

### 4.1 Adopting ONT long-read transcriptomics in non-model systems

Illumina has been the technology of choice for RNAseq transcriptomics given high data quality and well-established bioinformatics pipelines (Conesa et al., 2016; Hölzer and Marz, 2019; Corchete et al., 2020). However, short CDS reads still need to be stitched together *in silico* using a reference database or *via de novo* concatenated assembly to create contiguous sequences (contigs), from which full-length, protein-coding transcripts are derived. In non-model organisms with no reference databases, partially or spuriously assembled CDS and chimeric transcripts are common pitfalls (Conesa et al., 2016; Freedman et al., 2021). Moreover, in evolutionarily closely-related genes and in gene families consisting of multiple isoforms, it is difficult to resolve CDS and quantify gene expression to isoform level using short-reads (Steijger et al., 2013; Conesa et al., 2016).

Recent inventions in Third Generation Sequencing technologies return high throughput, long-read sequences that provide us with end-to-end transcripts, thereby eliminating the need for assembling contigs. Oxford Nanopore Technologies (ONT), arguably the leader in long-read transcriptomics, offer short to ultra-long DNA/RNA molecules longer than 10 kbp in length. Single-use cartridges with pre-loaded reagents that can be easily used with portable, bench-top instruments makes ONT a convenient platform. Further, ONT offers cDNA sequencing, in both polymerase chain reaction (PCR) based and PCR-free formats, and direct RNA sequencing that bypasses the need for converting RNA into cDNA. Despite these advantages, a major obstacle to the adoption of ONT long-read transcriptomics is the high error rate reported in both cDNA and direct RNA sequencing (Workman et al., 2019). Moreover, although over 555 tools are available for long-read analysis (https://long-read-tools.org/) (Amarasinghe et al., 2020; Amarasinghe et al., 2021), no clear bioinformatics pipelines have been established for *de novo* ONT transcriptomics and we are still in early stages of reference-free ONT transcriptomics. One way to mitigate sequencing errors is by adopting a reference-based approach, and as mentioned earlier, well-characterized, species-specific reference databases can go a long way in resolving transcript sequences and their expression levels, even to the level of isoforms (Sessegolo et al., 2019; Workman et al., 2019; Dong et al., 2021). This approach with ONT is often used in species with good quality reference databases such as humans (Weirather et al., 2017; Soneson et al., 2019; Workman et al., 2019), mice (Sessegolo et al., 2019), cattle (Halstead et al., 2021), fruit flies (Bayega et al., 2018), viruses (Boldogkői et al., 2018), and well-studied plants (Cui et al., 2020; Wang et al., 2021). However, in non-model species without reference databases, the uptake of ONT long-read transcriptomics is still not widespread.

We present the first study to adapt ONT long-read RNAseq in a completely *de novo* and reference-free approach to characterize novel genes in any animal species. Our study shows that ONT can be successfully used to sequence mRNA transcriptomes of even minute tissues such as insect AGs, with more than two dozen novel AG genes discovered from *S. punctum* (Figure 4B). Thus far, novel insect reproductive genes and gene products have been primarily discovered using Illumina based RNAseq and microarrays (Vibranovski et al., 2009; Sayadi et al., 2016; Bayram et al., 2017; Vedelek et al., 2018; Mrinalini et al., 2021), as well as using traditional protein or proteomics analyses (Peferoen and De loof, 1984; Parthasarathy et al., 2009; Goenaga et al., 2015; Gorshkov et al., 2015; Wei et al., 2015; Yamane et al., 2015). Gene sequences from our assembly-free and reference-free ONT long-read pipeline were of high quality, with sequence similarity levels comparable to genes derived from Illumina short-read assembly (Table 2; Figures 5B,C). Error-prone ONT long-reads, when combined with effective read quality filtering and rigorous error correction methods, can be a reliable new technology for end-to-end gene discovery and expression quantification that eliminates the need for transcriptome assemblies or reference databases. Moreover, gene expression quantification for ONT was highly congruent with Illumina (Figures 4A,D,E). Finally, we were able to achieve results comparable to Illumina without the need for transcriptome assembly, and using only a quarter the number of sequenced bases than that of Illumina sequencing (Table 1). ONT long-reads can therefore be a reliable, resource-friendly, and costeffective alternative that can achieve end-to-end sequencing of novel genes from non-model species, even in the absence of a reference database.

### 4.2 *De novo* gene discovery and gene expression quantification in a non-model species: ONT GridION v/s Illumina

Our *de novo* ONT transcriptome pipeline, incorporating read quality filtering and rigorous post-sequencing error correction procedures, successfully mitigated high error rates in ONT long-reads (Figure 2A, 4B) (Sahlin and Medvedev, 2019; Sahlin et al., 2021). Using ONT GridION, we successfully discovery novel AG genes, which constituted 65% of 40 high-quality and high-confidence gene set and cross-verified against evidence from Illumina transcriptomes (Figure 4B). In our analysis of sequence similarity, median sequence similarities of genes and proteins in ONT (to their respective orthologs in the Illumina control sample) are comparable to within-Illumina median sequence similarities (Table 2). Further, although two different methods of gene expression quantification were used for ONT and Illumina pipelines (Figure 2A), there was a high degree of congruence between the two technologies with respect to overall expression pattern (Figure 4A) and in terms of proportion of expression in each functional category (Figures 4D,E). At the level of individual *S. punctum* genes, expression patterns were less congruent, and could be attributed to variation in biological replicates (Figure 5A).

When comparing sequences across technologies at the nucleotide level, ONT samples (ONT-1 and ONT-2) had 53% and 50% sequences that were 100% similar to Illumina control orthologs (Table 2). At the protein level, ONT samples show 43% and 45% of sequences are 100% similar to the Illumina control orthologs (Table 2). This is comparable to within Illumina assessment, where 65% of nucleotide sequences and 43% of protein sequences were found to be 100% similar to each other among the two samples. Due to the small size of *S. punctum* and the minute size of our tissue, our samples were collected by pooling tissues dissected from multiple flies collected at different time points. Therefore, the lack of 100% sequence similarity among a majority of sequences even within Illumina suggests that rather than sequencing errors, natural genetic variation together with differential representation and incorporation of transcripts during *de novo* transcriptome construction could be the likely source of sequence variation. This is corroborated by the much larger proportion (over 90%) of genes that are 100% similar within ONT at both nucleotide and protein levels. ONT uses a consensus calling and polishing approach which could iron out high levels of differences, be it sequencing errors or sequence variation.

In order to find genes that are uniquely identifiable in either ONT or Illumina, we investigated samples within each technology. We found seven transcripts in total, of which six were uniquely present in ONT samples but not in Illumina, and one was uniquely present in Illumina samples but not in ONT. Among transcripts identified only in ONT, we found two potential genes, and both of these were 100% identical at the protein level when compared across the two samples. However, the functional relevance of both these genes is unclear as they do not show similarity to any other proteins in *nr,* they do not contain a signal peptide, and they do not show high expression levels in AGs of *S. punctum* either. Of the remaining four transcripts unique to ONT, one was found to be a chimera of poly-AAA tail sequence that had been missed by the filter. Three other transcripts showed poor sequence similarity, and were incongruent between samples either throughout the length of the sequence or at C-terminal ends of their protein sequence, therefore making identification of the correct sequence unreliable. In the single gene unique to Illumina samples, the C-terminal was partially absent in one of the samples and the functional significance was not clear in this case either.

### 4.3 Evolution of novel reproductive genes

We successfully discover 26 novel genes that are expressed in the male AGs of *Sepsis punctum* (Figure 4B)*.* These novel AG genes are also of *unknown function* as we find no homology to any other proteins in *nr*. However, these genes are among the highest expressed in AG tissues, both as a gene category (81% of total expression) (Figure 4C) and at the level of individual genes (Figure 5A). Novel genes with high expression that also encode seminal fluid proteins may play a role in AG function and *S. punctum* reproduction (Figure 4C). Our results are similar to patterns of gene evolution and gene expression that have been reported in other insect species (Parthasarathy et al., 2009; Sayadi et al., 2016; Bayram et al., 2017; Gotoh et al., 2018; Bayram et al., 2019; Mrinalini et al., 2021). Much of what we know about the mechanisms underlying insect reproductive gene evolution comes from model species like *Drosophila melanogaster* (Chen et al., 2013; Sirot, 2019). It is generally accepted that novel genes often arise *via* neo- or sub-functionalization following duplication of existing protein coding genes (Wagstaff and Begun, 2007; Mancini et al., 2011; LaFlamme et al., 2012; Sirot et al., 2014; Garlovsky et al., 2020). However, novel genes may also evolve *de novo* from non-coding regions of a genome, and several studies have suggested that insect reproductive tissues are candidate sites for the recruitment of *de novo* evolved genes (Levine et al., 2006; Begun et al., 2007; Reinhardt et al., 2013; Mrinalini et al., 2021; Rivard et al., 2021). Novel AG genes have been shown to evolve and diversify rapidly through genomic innovation followed by recruitment for high expression in insect AG tissue (Mrinalini et al., 2021), as well as through rapid evolution at the primary sequence level (Findlay and Swanson, 2010; Avila et al., 2011).

Despite such evidence, the selective forces driving this rapid evolution and diversification of reproductive genes are not always well understood. For instance, adaptive evolution and positive selection have often been invoked to explain this phenomenon in various animal species (Birkhead and Pizzari, 2002; Swanson and Vacquier, 2002; Torgerson et al., 2002; Jansa et al., 2003; Teng et al., 2017; Weber et al., 2017; Rowe et al., 2020). Specifically, adaptive evolution *via* post-copulatory sexual selection predicts strong directional selection for fast-evolving reproductive genes that would increase competitive fertilization among ejaculates or mediate ejaculate-female interactions. Molecular evolutionary studies comparing dN/dS rates of reproductive genes and non-reproductive genes among closely-related species also suggest that this can drive divergence between species (Ahmed-Braimah et al., 2017; Rowe et al., 2020). Others suggest that positive selection can be related to immune functions because seminal fluids may have antibacterial properties (Mueller et al., 2007; Wong et al., 2008). However, there is also evidence that most reproductive genes may not evolve adaptively since less than 12% of seminal fluid proteins in *D. melanogaster* actually display evidence of positive selection and that both slow *and* fast evolving genes have been found to be functionally important for insect reproduction (Findlay et al., 2008; Findlay and Swanson, 2010; Hurtado et al., 2021; Patlar et al., 2021). Therefore, relaxed selection has been proposed as an alternative hypothesis given that the expression of reproductive genes is sex-specific and selection is also limited to each sex (Dapper and Wade, 2020). Studies that assess dN/dS taking into account polymorphisms or variation at the population level suggest that more than 50% of AG genes are in fact under relaxed selection (Patlar et al., 2021). *Sepsis punctum* populations in North America and Europe exhibit stark variation in both female remating rates as well as male reproductive investments (Puniamoorthy et al., 2012a; Rohner et al., 2016), and comparative molecular evolutionary analysis across multiple populations may provide interesting insights on the selective forces behind this rapid evolution and diversification of reproductive genes.

### 4.4 Role of *protease inhibitors*, *C-type lectins*, and *cystatins*

In addition to novel genes, *S. punctum* AG transcriptomes revealed ten protease inhibitors and two genes each of C-type lectins and cystatins (Figure 4C). *Protease inhibitors* were the largest group and the second highest expressed functional category of genes in *S. punctum* (Figures 4B,C). Consisting of a large and diverse group of genes that synthesize many classes of proteins, *protease inhibitors* have been found in seminal fluids of *D. melanogaster* (Swanson et al., 2001; Mueller et al., 2008). They play a role in male sperm competitiveness and protect seminal fluids and sperm from proteolysis in the sperm storage organs of female flies post-mating (Park and Wolfner, 1995; Mueller et al., 2008). *C-type lectins* are immune-related genes that have been found in many insects that could be involved in protecting seminal fluids from microbial infections (Tian et al., 2017). *Cystatins* are also a large and diverse group of genes, that regulate the activity of cysteine and serine proteases. Although little is known about their specific functions in male reproductive tissues, *cystatins* have been found in seminal fluids and reproductive tissues of flies, flatworms and ticks (Sonenshine et al., 2011; Geadkaew et al., 2014; Garlovsky et al., 2020). They may be involved in spermatogenesis and fertilization (Geadkaew et al., 2014), and have been found to play a critical role in regulating programmed cell death during embryogenesis in plants (Zhao et al., 2014).

### 4.5 Genes encoding secretory proteins

Eighty percent (32) of AG genes analysed in *S. punctum* were likely to encode secretory proteins (Figure 4D). All 10 *protease inhibitors* and both *C-type lectins* and *cystatins* contain secretory signals, whereas among 26 novel *S. punctum* genes, 69% (18) encoded secretory proteins. These genes also accounted for the majority of AG gene expression, with an average of 84% of total expression in the four samples (Figure 4E). These patterns observed in *S. punctum* were similar to those found in other insects such as dung beetles, where 73% of AG genes analysed likely encoded secretory proteins, accounting for over 80% of total gene expression (Mrinalini et al., 2021). This supports that the primary function of male AG in most male insects is the synthesis of secretory proteins.

## 5 Conclusion

We discovered 26 novel reproductive genes that show high expression in the accessory glands of male *S. punctum.* We find that by implementing rigorous post-sequencing error correction procedures, error-prone ONT long-reads can produce gene sequence and gene expression data that are comparable to Illumina. Our study demonstrates that *de novo* ONT long-read transcriptomics is a reliable approach for novel gene discovery and gene expression analysis in the absence of reference databases. Gene discovery in non-model insects has important implications for understanding fundamental evolutionary processes such as phenotypic trait evolution, adaptation, and speciation. In particular, male reproductive genes of insects are known to synthesize seminal fluid proteins that interact with the female reproductive environment and thereby play a role in post-copulatory sexual selection. Hence, understanding rapid specialization and diversification of male reproductive genes in a species helps shed light on mechanisms of divergence of populations and the processes of incipient speciation.

## Data Availability

The datasets generated and analysed during the current study are available in GenBank at https://www.ncbi.nlm.nih.gov/and can be accessed with Project ID: PRJNA765219.

## Abbreviations

AG, Accessory Gland; cDNA, Complementary DNA; ONT, Oxford Nanopore Technologies; PacBio, Pacific Biosciences; PCR, Polymerase Chain Reaction; SMRT, Single-Molecule Real Time; TPM, Transcript per Million.
